# Validation of the Santa Clara Ethics Scale (SCES) in Nursing Students: The Role of Ethics as a Protector of Student Compassion

**DOI:** 10.3390/nursrep14040265

**Published:** 2024-11-21

**Authors:** Gabriel Vidal-Blanco, Javier Sánchez-Ruiz, Laura Galiana, Antonia Pades, Noemí Sansó

**Affiliations:** 1 Advanced Research Methods Applied to Quality of Life Promotion—ARMAQoL, Department of Nursing, University of Valencia, 46010 València, Spain; gabriel.vidal@uv.es; 2 Advanced Research Methods Applied to Quality of Life Promotion—ARMAQoL, Department of Methodology for the Behavioral Sciences, University of Valencia, 46010 València, Spain; jasan4@alumni.uv.es (J.S.-R.); laura.galiana@uv.es (L.G.); 3 Department of Nursing and Physiotherapy, University of Balearic Islands, 07120 Palma, Spain; antonia.pades@uib.es; 4 Balearic Islands Health Research Institute (IDISBA), 07120 Palma, Spain

**Keywords:** ethics, validation study, factor analysis, nursing students

## Abstract

Background: Ethics is one of the essential concepts associated with professional nursing practice, and can play a part in the development of compassion. Although a number of instruments have been developed for the measurement of ethics, most are context-specific or excessively lengthy. The Santa Clara Ethics Scale (SCES) overcomes these problems. The goal is to translate, adapt, and validate the Spanish version of the Santa Clara Ethics Scale and to study the role of ethics, as a moral resource, in the prediction of the levels of compassion of nursing students. Methods: This study is a translation, adaptation, and validation study, with a cross-sectional design. A total of 924 Spanish nursing students participated in this study. Ethics and compassion for others were measured. Analyses included a confirmatory factor analysis, reliability estimates, and a structural equation model in which ethics explained the five correlated dimensions of compassion for others. Results: The confirmatory factor analysis had an adequate fit: *χ*^2^(35) = 173.56 (*p* < 0.01), CFI = 0.94, TLI = 0.92, RMSEA = 0.07 [90% CI = 0.06, 0.08], and SRMR = 0.05. Internal consistency was adequate (α = 0.74; ω = 0.83). The predictive model pointed to positive and statistically significant relationships between ethics and all dimensions of compassion for others. Conclusions: The SCES can be considered a suitable instrument for the assessment of ethics in the Spanish nursing population and, thus, can be used as a tool for the measurement of key ethical competencies during the nursing degree. Moreover, the development of ethics is likely to improve the compassion levels of students. Ethics is, then, a key internal resource for both nursing students’ compassionate care skills and, consequently, must be taken into account when redefining nursing students’ curricula.

## 1. Introduction

Although the concept of ethics is almost as old as humanity itself, and it has been evolving together with us, it is commonly accepted that ethics is essentially the “principles of living that attempt to answer the ongoing and age-old question, ‘Who do you want to be?’” [1]. Another way of stating this concept is to say that ethics aims to give a systematic account of our judgments about conduct, in so far as these estimate it from the standpoint of right or wrong, good or bad [2].

Therefore, ethics is intimately linked to moral philosophy involving the examination of morality through a variety of different approaches [3], and it is especially important in settings requiring decision-making associated with important consequences.

It is not surprising, then, the international recognition of ethics as a key part of the work of nurses. There are commonly accepted principles in bioethics that include autonomy, beneficence, non-maleficence, and justice [4,5,6], which permeate nursing practice. Ethics is, therefore, one of the essential concepts associated with professional nursing practice. The study of ethics as it relates to nursing practice has led to the exploration of other relevant concepts, including moral distress, moral hazard, moral community, and moral or critical resilience [7,8,9,10].

This also makes ethics key when researching student nurses. The American Association of Colleges of Nursing, for example, recommends the inclusion of strategies to improve ethical thinking in nursing curricula [11]. In addition, some authors have pointed out the role that moral resources can play in the development of compassion. In this sense, the authors of Ref. [12] had already pointed out how moral resources are an elemental factor in the compassion of health professionals. According to these authors, moral resources would form part of the internal resources that make up the compassionate core of professionals, which is the basis from which altruistic acts towards others emanate [12]. Indeed, and according to Ozawa-de Silva et al. [13], ethics can be understood as a way of conceptualizing how human beings relate to one another and their environment with specific regard to suffering and its alleviation. As compassion involves an approach and recognition of others, and it is tied to a commitment and desire to relieve suffering, appropriate levels of ethics could thus contribute to improving the compassion levels of health professionals in general, and nursing students in particular. Therefore, the measurement and assessment of ethics in nurses and nursing students may be key to achieve compassionate care.

For this measurement of ethics, several instruments have been developed. Notable, for example, is the Multidimensional Ethics Scale (MES) [14], an eight-question ethics questionnaire that is mainly used in business settings, since the scale focuses primarily on the ethical decision-making process and provides respondents with business scenarios. Another instrument is the Measure of Moral Orientation (MMO) [15], which highlights social justice issues. However, this instrument is time-consuming to administer, as it uses 78 questions. On the other hand, the Defining Issues Test [16] is a measure of moral judgment based on Kohlberg’s stages of moral development, and includes dilemmas along with 12 issues that participants rank by importance, which aims to assess moral development along the cognitive progression. More recently, newer and more nursing-centered instruments have begun to appear; one of such is the revised Moral Sensitivity Questionnaire (MSQ) [17], which has nine items and assesses the nurse’s ability to sense the patient’s needs and their sense of responsibility to provide care for them. In this same line of thought, a new development, the Ethical Sensitivity Questionnaire for Nursing Students can be found (ESQ-NS) [18]. This test, which uses a pool of 13 items and specifically measures judgment of the care conflict and the sense of moral burden that nurses may encounter when working, leaves out the nurses’ ability to display compassion and their confidence on the decisions that need to be taken during ethical conflicts.

Considering problems such as the specificity or length of these scales, which makes them impractical for assessing more general ethics, Plante and McCreadie [1] developed the Santa Clara Ethics Scale, an ethics questionnaire of only 10 items, which can be used among college-age emerging adults. In previous studies, the scale has offered evidence of a single-factor structure, with adequate reliability estimates (α = 0.83). This scale is a general instrument of ethics, not focused on specific contexts (such as the MES, which is mostly used in business settings) and not including specific items for nursing practice such other instruments (i.e., MSQ, ESQ-NS), which prevent them from being used with other health professionals, such as physicians or psychologists, and also in students without practice experience. It is also better than others in terms of length, when compared to general instruments, such as the MMO. However, no Spanish version of the scale is available.

### Aim of the Study

In this context, the aim of the present study is to translate, adapt, and validate the Spanish version of the Santa Clara Ethics Scale and to study the role of ethics, as a moral resource, in the prediction of the levels of compassion of nursing students.

## 2. Materials and Methods

### 2.1. Translation and Adaptation Procedure

For the translation of the scale, we used the forward and backward translation process. First, the scale was translated into Spanish by a professional native; it was then translated back into English by another native professional. The final version was revised by four experts in Psychometrics and cross-cultural instrument development and validation, Educational Psychology and Developmental Psychology, and Nursing. All of them judged the instrument to adequately measure ethics, and no revisions from the original backward–forward translation were made. The Spanish version of the Santa Clara Ethics Scale is displayed in Table 1.

### 2.2. Study Design

This study is a translation, adaptation, and validation study, with a cross-sectional design. Data were gathered in May 2022, May 2023, and May 2024. In each year or moment of data collection, different individuals were sampled: 2022 first year nursing students, 2023 first year nursing students, and 2024 first year nursing students.

### 2.3. Setting and Participants

First year nursing students at the University of Valencia and the University of the Balearic Islands (Spain) were encouraged to participate. They were identified via the universities’ lists of registered students. In order to be included, participants had to be nursing students in their first year of their degree. The students completed the questionnaire online in approximately 20 min.

To determine the required sample size, the total population of the first year of the Degree in Nursing in academic years 2022, 2023, and 2024 at both universities was considered. For a population that was calculated to be N = 1296 (N = 432 each year), with a confidence interval of 95% and an error limit of 5%, the number of elements to obtain was n = 297.

### 2.4. Measures

For this work, the Spanish version of the Santa Clara Ethics Scale and the Sussex-Oxford Compassion Scale-Other were used. The Santa Clara Ethics Scale (SCES) [1] is a scale composed of 10 items that assess ethical commitment and interest. The items, which are answered in Likert-type format with four alternatives, from 1 (strongly disagree) to 4 (strongly agree), reflect ethical decision making and highlight a virtue and value approach to ethics that emphasizes respect, responsibility, integrity, competence, and concern for others. The theoretical model underlying the items comes from a list of virtues discussed in moral philosophy [19,20]. The psychometric properties of the scale are reported in Section 3.

The Spanish version of the Sussex-Oxford Compassion for Others Scale (SOCS–O) was used [21]. The SOCS–O assesses five dimensions of compassion for others from 20 items, four each: (a) recognizing suffering, (b) understanding the universality of suffering, (c) feeling compassion for the suffering person, (d) tolerating uncomfortable feelings, and (e) motivation to act/act to alleviate suffering [22]. Items score in a 5-point Likert-type scale ranging from 1 (not at all true) to 5 (always true). Reliability estimates in this study were: α = 0.867 and ω = 0.869 for Recognizing suffering; α = 0.865 and ω = 0.866 for Understanding the universality of suffering; α = 0.801 and ω = 0.821 for Feeling for the person suffering; α = 0.722 and ω = 0.758 for Tolerating uncomfortable feelings; and α = 0.879 and ω = 0.880 for Acting to alleviate suffering.

### 2.5. Data Analyses

Firstly, data analyses included percentages and frequencies for sample characterization.

In the second place, descriptive statistics for the items and the total scores of the Spanish version of the SCES were calculated. These included mean, standard deviation, minimum, and maximum scores.

Thirdly, for the study of the internal structure, a confirmatory factor analysis was hypothesized, estimated, and tested. The hypothesized model comprised one factor of ethics, which explained the ten items of the scale. To assess the model fit, several criteria were used: the chi-squared statistic, the Comparative Fit Index (CFI), the Tucker Lewis Index (TLI), the Root Mean Square Error of Approximation (RMSEA), and the Standardized Root Mean Square Residual (SRMR). CFI and TLI values above 0.90 (better over 0.95) and SRMR and RMSEA values below 0.08 (better under 0.06) were indicative of a good fit [22,23,24].

Then, estimates of reliability were gathered. These included internal consistency estimates for the items (homogeneity and alpha if item deleted) and the scale (Cronbach’s alpha and McDonald’s omega). For reliability estimates, values of 0.70 were considered adequate [25].

Finally, criterion-related validity evidence was gathered by studying the predictive power of the SCES scores on nursing students’ levels of compassion for others, whilst controlling for gender and age. For this purpose, a structural equation model was hypothesized, estimated, and tested, in which gender, age, and a latent factor of ethics, as measured with the Santa Clara Ethics Scale, predicted five correlated factors of compassion for others, measured by the Sussex-Oxford Compassion for Others Scale. The impacts of gender and age on ethics and the five dimensions of compassion for others were also estimated. To assess the model fit, the indices mentioned above were used, together with an examination of the relations in the model.

All of the models were estimated using weighted least squares mean and variances (WLSMV), that is, robust WLS. This method is recommended for ordinal and non-normal outcome variables, such as the one under study [26]. Indeed, simulation studies have indicated that CFA with polychoric correlations (1) is adequate under violation of the explicit, theoretical assumption that variables underlying polychoric correlations be normally distributed; and (2) provides stable and accurate estimations of model parameters, test statistics, and standard errors, under either bivariate normality or bivariate non-normality for latent response variables [27].

Therefore, all of the models were estimated using maximum likelihood with robust standard errors. This is the recommended option for ordinal and non-normal outcome variables, such as the one under study [26].

For the statistical analyses, SPSS version 28 [28] and Mplus version 8.4 [29] were used.

## 3. Results

### 3.1. Sample Description

The sample consisted of 924 nursing students, of which, 768 were female (83.12%). The mean age was 22.5 years (SD = 7.63). Of these, 615 (66.5%) participants were studying at the University of Valencia, while 309 were studying at the University of the Balearic Islands. Most of the students were not working at the time of the survey (72.3%). For more information, see Table 2.

### 3.2. Items and Scale’s Descriptive Statistics

The descriptive statistics of the Santa Clara Ethics Scale items showed medium-high scores, ranging from 2.32 (item 9) to 3.63 (item 1). The mean score on the scale was 3.30 (SD = 0.38). All items showed negative skewness, apart from item 9 (0.205). Kurtosis indices showed that items ranged between mesokurtic and leptokurtic, with only item 7 categorizing as platykurtic. Finally, all items showed evidence of non-normality. These results can be seen in Table 3.

The scores on compassion for others, students’ levels were high, ranging from 3.97 (SD = 0.66) for the Recognizing suffering dimension, 4.06 (SD = 0.61) for Tolerating uncomfortable feelings, 4.24 (SD = 0.64) for Feeling for the person suffering, 4.33 (SD = 0.63) for Acting to alleviate suffering, to 4.55 (SD = 0.59) for Understanding the universality of suffering. Overall, the mean in compassion for others was 4.23 (SD = 0.53). All items showed negative skewness. Kurtosis analyses showed that most of the items ranged from mesokurtic to leptokurtic, with only items 11, 18, and 19 being platykurtic. Furthermore, all items showed evidence of non-normality.

### 3.3. Confirmatory Factor Analysis

**Figure 1:**
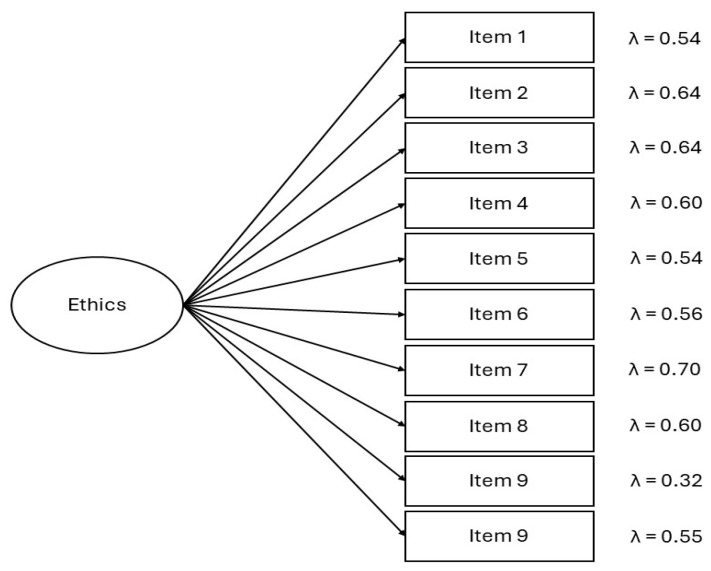
CFA for a single factor of Ethics for the Santa Clara Ethics Scale.

The confirmatory factor analysis, in which a single latent factor of ethics explained all 10 items of the scale, had an adequate fit: *χ*^2^(35) = 173.56 (*p* < 0.01), CFI = 0.94, TLI = 0.92, RMSEA = 0.07 [90% CI = 0.06, 0.08], and SRMR = 0.05. Factor loadings for all of the items were statistically significant (*p* < 0.01), ranging from 0.32 (item 9) to 0.70 (item 7). These results can be seen in Figure 1.

### 3.4. Reliability Estimates

Items’ evidence of reliability showed adequate estimates of item–total correlations and reliability: item–total correlations were in the range of 0.49 (item 9) to 0.64 (item 7), above the acceptable minimum of 0.30 [30] and the removal of any of the items supposed a significant decrease in the reliability estimate of the scale (see Table 3). The reliability coefficients of the scale were also adequate, with α = 0.74 and ω = 0.83.

### 3.5. Criterion-Related Validity Evidence: Prediction of Compassion Through Ethics

**Figure 2:**
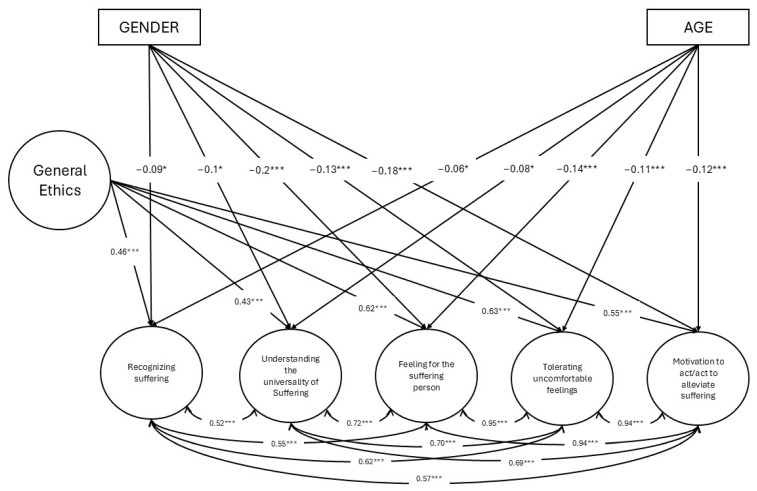
Predictive capacity of ethics over compassion whilst controlling for age and gender. Notes: * *p* < 0.05; *** *p* < 0.001.

Finally, we tested a predictive model in which a general factor of ethics predicted nursing students’ levels of compassion for others, whilst controlling for gender and age. The model showed an excellent fit: *χ*^2^(438) = 1210.89 (*p* < 0.01), CFI = 0.98, TLI = 0.97, RMSEA = 0.05 [90% CI = 0.04, 0.05], and SRMR = 0.04. Results of the model can be consulted in Figure 2.

Regarding the psychometric part of the tested model, all factor loadings approached acceptable levels and showed the expected directions. In the case of the SCE-S, all of them surpassed the 0.50 mark but for item 9, with loadings ranging from λ = 0.71 for item 5 to λ = 0.35 for item 9. This same phenomenon was seen throughout the factorial loadings of the SOCS-O, with all items surpassing the 0.50 threshold but for item 4 of the Tolerating uncomfortable feelings dimension. For the SOCS-O items, the weights varied from λ = 0.92 for item 2 of the Recognizing suffering subscale to λ = 0.39 for item 4 of the Tolerating uncomfortable feelings dimension. All of these results can be consulted in Table 4.

Regarding the predictive part of the model, results pointed to positive and statistically significant relationships between ethics, measured with SCE-S, and all dimensions of compassion for others: Recognizing suffering (*β* = 0.46, *p* < 0.001), Understanding the universality of suffering (*β* = 0.43, *p* < 0.001), Feeling for the suffering person (*β* = 0.62, *p* < 0.001), Tolerating uncomfortable feelings (*β* = 0.63, *p* < 0.001), and Motivation to act/act to alleviate suffering (*β* = 0.55, *p* < 0.001). The controlled variables, gender and age, did not exert significant effects on the general level of ethics of the students; nevertheless, both of them showed significant predictive power over the five compassion for others subscales. On the one hand, gender displayed negative and small-to-medium predictive relationships with every one of the aforementioned subscales, with its strongest effect being noted over the ‘Feeling for the suffering person’ subscale (*β* = −0.2, *p* < 0.001) followed by ‘Motivation to act/act to alleviate suffering’ (*β* = −0.18, *p* < 0.001), then ‘Tolerating uncomfortable feelings’ (*β* = −0.13, *p* < 0.001), then ‘Understanding the universality of suffering’ (*β* = −0.1, *p* < 0.05), and its weakest effect over the ‘Recognizing suffering’ (*β* = −0.09, *p* < 0.05) dimension. As women were codified as 0 and men as 1, being a man predicted lower levels of compassion for others throughout all its dimensions. On the other hand, age had little to no effect on the previously cited dimensions, showing significant but very small relationships with them, the strongest of them being with ‘Feeling for the suffering person’ (*β* = −0.14, *p* < 0.001), followed by ‘Motivation to act/act to alleviate suffering (*β* = −0.12, *p* < 0.001), then ‘Tolerating uncomfortable feelings’ (*β* = −0.11, *p* < 0.001), then ‘Understanding the universality of suffering’ (*β* = −0.08, *p* < 0.05) and, lastly, ‘recognizing suffering’ (*β* = −0.06, *p* < 0.05).

## 4. Discussion

This study aimed to translate, adapt, and validate the Spanish version of the Santa Clara Ethics Scale proposed by Plante and McCreadie [1] and to study the role of ethics, as a moral resource, in the prediction of the levels of compassion of nursing students. In this sense, our specific aims sought to evaluate the SCE-S’s internal factor structure, its reliability, and improve upon the previously published evidence of its criterion validity. To achieve these goals, reliability analyses, a confirmatory factor analysis (CFA), and a structural equation model were used.

First, seeking to assess the SCE-S’s construct validity, we conducted a confirmatory factor analysis (CFA) to test the fit of a one-factor structure of general ethics on the data. The results showed a great fit for this model, as all fit indices surpassed the a priori imposed thresholds. Moreover, all factor loadings surpassed the mark of 0.50 but for item 9 “I would not be embarrassed if all of my actions were filmed and played back for others to see and evaluate”. The distinct differential functioning of this precise item might be due to the fact that the respondents are on their first year of undergraduate studies and, hence, feel they still have much to learn before being models or, at least, being recorded for their performance. It could also be hypothesized that item 9 shows a different behavior from the rest insofar as it includes content referring to privacy and public judgment. It may be that the fact that the item contains the facets of being recorded or feeling observed and judged by others, being the only item that collects this type of content, makes the item behave differently from the other items.

Secondly, the reliability analyses also demonstrated an acceptable level of accuracy of the items, as the scale also exceeded the selected cut-off points for both Cronbach’s Alpha and McDonald’s Omega. Specifically, values were similar to those reported in business measures, such as the MES or the ESQ-NS. Regarding the MSQ, no reliability estimations were provided in the original work [17]; the one provided for the Korean validation [31] was referred to the whole scale, whereas a multidimensional structure was defended. Factor estimations in the Chinese validation [32] were similar to the ones found in the current study for the SCES. Moreover, as regards the SCES, all items contributed positively to the general consistency of the scale, with indices showing reductions when taking away each item.

Thirdly, evidence of the criterion validity of the SCE-S was gathered through the aforementioned structural equations model, which showed that the level of ethics of Spanish nursing students can account for great amounts of the variability of the different subscales of the compassion for others construct. Different studies have also pointed out this relationship, where ethics is shown as a great predictor of compassion [1,33]. Moreover, other studies suggest that this relationship can be understood the other way around, as compassion is a key ability to understand others and to participate in acts to alleviate their suffering [22,34,35,36,37], these being central problems to the development and practice of ethics [13]. In any case, the relationship between ethics and compassion is clearly stated, being moral resources not only important for the nursing profession, but also an elemental factor for altruistic acts toward others, which are part of the nursing profession.

When examining the effects of gender and age, the obtained results suggest that both of them play a significant role in the prediction of compassion. In this sense, both of them exert a negative and direct effect on all of the dimensions of the SOCS-O (i.e., Recognizing suffering, Understanding the universality of Suffering, Tolerating uncomfortable feelings, Feeling for the suffering person, and Motivation to act/act to alleviate the suffering), with gender displaying small-to-medium effects, and age only small ones.

As these relationships between gender and the SOCS-O were negative and women were coded with “0” and men with “1”, they are signs that women had greater compassion for others. In the past, other studies have encountered this same phenomenon, with results pointing out greater compassion for others scores in women in contrast to men [21,22,38,39].

When it comes to the effect of age, the results pointed out that, at least minimally, it had an inverse relationship with the levels of compassion for others, with older students displaying smaller scores. Although contradictory, this finding has also been previously reported throughout scientific literature, more specifically in the context of Spanish nursing students [20]. In Kret’s study [40], younger nurses were perceived as more compassionate than older nurses across a patient sample. Moreover, this same trend has been present in the literature on the relationship between ethics and age, with older professionals displaying lower levels of ethical sensitivity [41]. Outside the healthcare context, a decrease in compassion has also been observed with age, but in adolescent samples [42]. In young adults, compassion has proved to increase [43], as those with older age are abler to recognize the universality of suffering and better able to maintain a mindful perspective [40]. However, these results refer specifically to self-compassion. So, results regarding age should be interpreted with caution, since the sample was composed of first-year nursing students, the variability of age was low, and most of the students were very similar in age.

The relationship between ethics and compassion is central to nursing, as ethics helps nurses deliver compassionate, patient-centered care [44]. The ability to empathize with patients, understand their suffering, and take action to alleviate it directly aligns with the ethical principles of respect for persons, beneficence, and justice. Compassion is often the result of a moral framework that encourages healthcare professionals to go beyond technical proficiency and engage with patients on a human level, offering not only physical care, but emotional and psychological support, the foundation of compassionate care. [45]. Scientific evidence suggests that fostering compassionate care in nursing education can enhance ethical sensitivity and resilience, equipping future nurses to handle complex interactions with patients more empathetically and effectively [46]. Therefore, integrating compassion-based training into ethics programs strengthens ethical reasoning, minimizes compassion fatigue, and improves the nurse–patient relationship [47].

This study has some strengths, such as the big sample size or its originality, being the first study to present the Spanish version of the SCES and to use it in a sample of nursing students. It is also one of the few that has empirically studied the relation between ethics and compassion for others. However, this study also has some limitations. One of its major shortcomings is the absence of a pilot study, in which we could have collected evidence of content validity. Future studies should, then, gather evidence on content validity, to better understand the functioning of particular items, such as the one of item 9.

## 5. Conclusions

In light of the results, the validation of the Spanish translation of the Santa Clara Ethics Scale (SCE-S) [1] has shown evidence of both a robust internal factor structure and criterion validity. For these reasons, it can be considered a suitable instrument for the assessment of ethics in the Spanish nursing population and, thus, can be used as a tool for the measurement of key ethical competencies during the nursing degree. Moreover, as the predictive model has shown, the development of ethics is likely to improve the compassion levels of students, which are also related to the quality of care provided and other positive outcomes [41]. Ethics is, then, a key internal resource for both nursing students’ and professional nurses’ compassionate care skills and, consequently, must be taken into account when redefining nursing students’ curricula. This dual focus on ethics and compassion is essential for the cultivation of empathetic, ethical, and high-quality nursing practice.

## Figures and Tables

**Table 1:** Spanish version of the Santa Clara Ethics Scale.

Item Num.	Item Content
1	Para mí, es muy importante respetar a los demás, incluso a los que no me gustan o con los que no estoy de acuerdo. [Respecting others, even those who I don’t like or agree with, is very important to me]
2	Para mí, es muy importante ser responsable y responder de mis actos, incluso cuando ello implica reconocer que no tengo razón o que he cometido un error. [Being responsible and accountable, even when I have to admit that I’m wrong or have errored, is very important to me]
3	Para mí, es muy importante ser sincero/a, justo/a e íntegro/a, incluso en situaciones en las que puede perjudicarme. [Being honest, fair, and maintaining integrity, even when it might put me at a disadvantage, is very important to me]
4	Me esfuerzo por ser competente a nivel personal y profesional y soy el primero/la primera en reconocerlo cuando no lo soy y no he dado la talla. [I strive to be competent in my areas of personal or professional expertise and am the first to admit it when I am not and have fallen short]
5	Siento mucha compasión por los demás, incluso por aquellos que no conozco o con los que tengo pocas cosas en común. [I feel a great deal of compassion for others, even those whom I don’t know or have few things in common with]
6	Tengo muy claros mis principios éticos y los sigo en todo momento. [I have clear ethical guiding principles that I keep in mind and follow at all times]
7	Para mí, en la vida es más importante actuar de manera ética que obtener algún beneficio personal. [It is more important for me to behave ethically than to get an advantage in life]
8	Nunca me aprovecho de los demás y soy sincero/a en mis relaciones e interacciones, incluso en situaciones en las que puede perjudicarme. [I never take advantage of others and am truthful in my relationships and interactions even when it might put me at a disadvantage]
9	No me daría vergüenza que grabaran y proyectaran todos mis actos y que los demás los vieran y los juzgaran. [I would not be embarrassed if all of my actions were filmed and played back for others to see and evaluate]
10	Antes de tomar una decisión, suelo preguntarme qué es lo correcto desde el punto de vista ético o moral. [I typically ask myself what the right thing to do is from an ethical or moral perspective before making decisions]

**Table 2:** Descriptive statistics for the sample.

Variables/Groups	n	%
Sex		
Women	768	83.12
Men	151	16.34
Missing	5	0.54
University		
University of Valencia	615	66.5
University of the Balearic Islands	309	33.5
Missing	0	0
Working Status		
Not working	668	72.3
Full-time	116	12.5
Part-time	136	14.7
Missing	4	0.05

**Table 3:** Descriptive statistics for the Spanish version of the Santa Clara Ethics Scale (SCE-S).

Items	M	SD	Min	Max	Sk	K	Lambda	Item-Total r	a_iid_	W
1	3.63	0.58	1.00	4.00	−1.56	2.64	0.54	0.52 ***	0.72	0.63 ***
2	3.62	0.57	1.00	4.00	−1.31	1.35	0.64	0.56 ***	0.72	0.65 ***
3	3.55	0.58	1.00	4.00	−0.98	0.56	0.64	0.57 ***	0.71	0.69 ***
4	3.45	0.62	1.00	4.00	−0.84	0.57	0.60	0.57 ***	0.71	0.73 ***
5	3.37	0.72	1.00	4.00	−0.95	0.46	0.54	0.57 ***	0.72	0.76 ***
6	3.42	0.60	1.00	4.00	−0.62	0.15	0.56	0.55 ***	0.72	0.73 ***
7	3.27	0.68	1.00	4.00	−0.51	−0.31	0.70	0.64 ***	0.70	0.79 ***
8	3.33	0.65	1.00	4.00	−0.61	0.05	0.60	0.60 ***	0.71	0.77 ***
9	2.32	0.96	1.00	4.00	0.21	−0.09	0.32	0.49 ***	0.76	0.88 ***
10	3.04	0.74	1.00	4.00	−0.46	0.00	0.55	0.58 ***	0.72	0.74 ***
Total score	3.30	0.38	1.00	4.00	−0.76	1.88	---	---	---	

Notes: *** *p* < 0.001.

**Table 4:** Factor loadings of items in their respective scales (SCE-S and SOCS-O).

Items	Ethics	Recognizing Suffering	Understanding the Universality of Suffering	Feeling for the Suffering Person	Tolerating Uncomfortable Feelings	Motivation to Act/Act to Alleviate Suffering
Item 1	0.58	0.83	0.83	0.83	0.84	0.88
Item 2	0.59	0.92	0.81	0.87	0.87	0.89
Item 3	0.57	0.80	0.91	0.90	0.76	0.88
Item 4	0.60	0.87	0.90	0.54	0.39	0.83
Item 5	0.71	--	--	--	--	--
Item 6	0.58	--	--	--	--	--
Item 7	0.62	--	--	--	--	--
Item 8	0.57	--	--	--	--	--
Item 9	0.35	--	--	--	--	--
Item 10	0.53	--	--	--	--	--

## Data Availability

The data that support the findings of this study are available from the corresponding author upon reasonable request.

## Public Involvement Statement

No public involvement in any aspect of this research.

## Guidelines and Standards Statement

This manuscript was drafted against the Strengthening the Reporting of Observational Studies in Epidemiology (STROBE) Statement for observational studies.
